# Occurrence, Distribution, and Risk of Organophosphate Flame Retardants in Sediments from Jiulong River Estuary and Adjacent Western Taiwan Strait, China

**DOI:** 10.3390/ijerph19042449

**Published:** 2022-02-20

**Authors:** Ling Cai, Yuwei Shi, Chenyuan Pan, Feng Zhu, Siqi Wang, Juanjuan Dai, Ming Yang, Jing Ma

**Affiliations:** 1 Third Institute of Oceanography, Ministry of Natural Resources, Xiamen 361005, China; cailing@tio.org.cn (L.C.); XJsyw9221@163.com (Y.S.); daijuanjuan@tio.org.cn (J.D.); 2 School of Environmental and Chemical Engineering, Shanghai University, Shanghai 200444, China; zhufeng666@i.shu.edu.cn (F.Z.); wsiqi@t.shu.edu.cn (S.W.); mingyang@shu.edu.cn (M.Y.)

**Keywords:** organophosphate flame retardant, sediment, risk assessment, Jiulong River estuary, western Taiwan Strait

## Abstract

Organophosphate ester flame retardants (OPFRs) are widely prevalent in the environment and are of significant concern because of their potential toxicity to human health and wildlife. In this study, the concentration, frequency, spatial distribution, potential sources, and ecological risks of OPFRs in sediments from the Jiulong River estuary and the adjacent western Taiwan Strait were investigated. Concentrations of four of the five studied OPFRs were between <LOD and 36.6 ng/g. The distribution of all OPFRs, except 2-Ethylhexyl diphenyl phosphate (EHDPP), remained highly consistent with hydrological (salinity) trends. Furthermore, a significantly positive correlation between EHDPP and total concentrations suggested that it may be the dominant contaminant at both sites. Principal element analysis indicated multiple sources of OPFRs, which were categorized as emissions from road runoff and surface traffic, effects of atmospheric deposition and hydrologic conditions, and a combination of industrial and population effects. Ecological risk indicates that tris (chloroethyl) phosphate (TCEP) and triphosphate ester (2,3-dibromopropyl) (TDBPP) have almost no risk, tris (clorisopropyl) phosphate (TCPP) generally has low risk, while EHDPP has moderate risk with the highest value of 0.487 in the sediments from both sites. Meanwhile, TCPP and TCEP exhibit lower theoretical health risks but are still not negligible. Overall, this work provides data to support global pollutant studies and facilitate the implementation of pollutant control strategies.

## 1. Introduction

Organophosphate ester flame retardants (OPFRs) are widely used as alternatives to brominated flame retardants in various commercial products, including furniture, textiles, electronics, vehicles, and petroleum, and for plasticizing, defoaming, and improving fire safety [1,2]. From 1992 to 2015, the global consumption of OPFR increased from 100,000 t to 680,000 t [1,3]. In 2007, the total OPFR consumption was approximately 85,000 t in Europe and 70,000 t in China, with an annual growth rate of 15% [4,5]. Moreover, OPFRs were detected in different environmental matrices, such as indoor dust, air, water, soil, and sediments [6,7,8,9,10]. Their long-distance mobility caused detectable OPFR pollution in Canada’s Arctic region and the central Arctic Ocean region [11,12]. Additionally, OPFR residues were detected not only in marine and freshwater biota but also in humans [13,14,15,16]. Due to the increasing demand for OPFRs by industries, their ubiquitous distribution in the environment has raised concerns about their potential toxicity and risk to human health and wildlife [17].

Previous studies showed that exposure to OPFR leads to adverse effects on animals and humans, including acute, reproductive, and developmental toxicity, neurotoxicity, organ toxicity, genotoxicity, mutagenicity, and endocrine disruption [18]. For example, tris (1,3-dichloroisopropyl) phosphate (TDCPP) affects liver cells and neurons, while tris (clorisopropyl) phosphate (TCPP) affects fertility [19]. In addition, OPFRs (especially water-soluble OPFR compounds) have high acute toxicity and endocrine-disrupting effects on aquatic organisms, such as algae, daphnia, and fish [20,21]. The U.S. Environmental Protection Agency (EPA) estimated that 80% of human exposure to OPFRs comes from indoor dust [15]. A population epidemiological study found that human exposure to TCEP via indoor dust was positively associated with papillary thyroid cancer [odds ratios = 2.42 (95% CI: 1.10, 5.33), *p* = 0.03] [17]. In the past decade, almost all produced OPFRs were detected in marine and freshwater animals. In Canada, multiple OPFRs were detected in lake trout (*Salvelinus namaycush*) and walleye (*Sander vitreus*) collected in different lakes, with detected concentrations ranging from <0.07 to 9.8 ng/g (WW) [13]. OPFRs enter the marine environment through human activities and accumulate in the ocean via rivers, lakes, and atmospheric deposition [20,22,23]. Therefore, it is important to determine the sources of OFPRs and their mode of distribution, migration, and environmental transformation to protect marine aquatic ecosystems.

Most studies on OPFRs tend to focus on dust, air, and surface water. A limited number of studies have focused on sediments, which are an important component of the marine environment and are closely related to aquatic fauna and ecological cycles [12,17,24]. In the Asia-Pacific region, OPFRs in sediments were primarily reported in the Yellow Sea, Bohai Sea, Laizhou Bay, Pearl River Delta, and offshore waters of Xiamen [17,23,25]. The Jiulong River estuary and adjacent western part of the Taiwan Strait have developed industries, high urbanization rates, and significant concentrations of halogenated flame retardants [17]. Although OPFRs are gradually being phased out, their hazards cannot be ignored [17,26,27]. In this study, we sampled inland estuaries and land–sea junctions of the aforementioned regions to study the content, distribution, pollution characteristics, and migration of OPFRs in sediments and analyzed their sources and potential risks.

## 2. Materials and Methods

### 2.1. Chemicals and Reagents

OPFR standards, including tris (chloroethyl) phosphate (TCEP), triphosphate ester (2,3-dibromopropyl) (TDBPP), tris (1,3-dichloroisopropyl) phosphate (TDCPP), 2-Ethylhexyl diphenyl phosphate (EHDPP), tris (clorisopropyl) phosphate (TCPP), and d15-triphenyl phosphate (d15-TPP) were purchased from Cambridge Isotope Laboratories Inc. (Andover, MA, USA). Detailed structures and physicochemical properties of the OPFRs are listed in Table S1. Acetonitrile, methanol, and formic acid (HPLC grade) were purchased from Anpel Laboratory Technologies Inc. (Shanghai, China). Ultra-pure water was produced using a Milli-Q water purification system (Millipore, Bedford, MA, USA).

### 2.2. Sampling Collection

**Figure 1:**
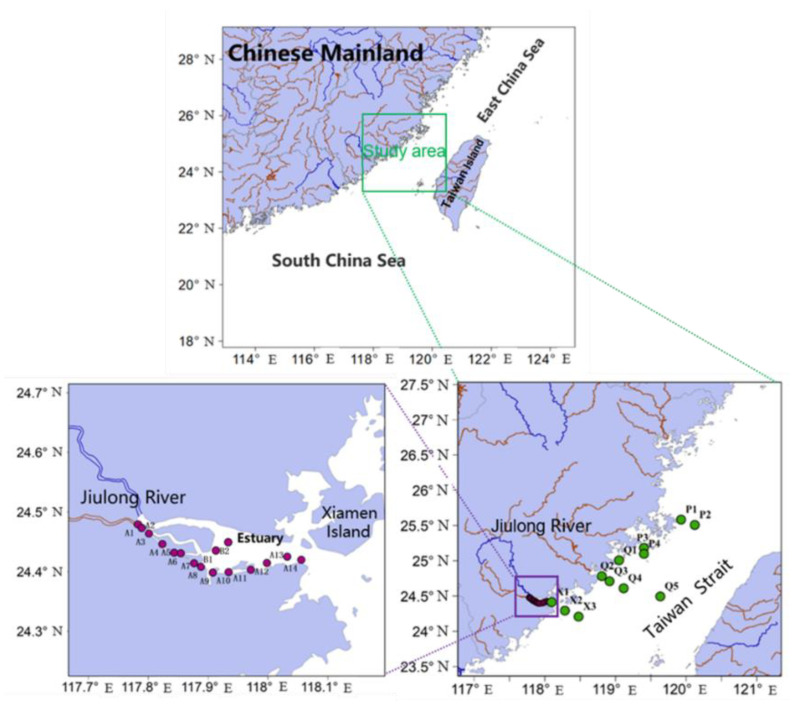
Sampling sites in the estuary of the Jiulong River and the western part of the Taiwan Strait.

The study area included the Jiulong River Estuary (JRE) and the adjacent western part of the Taiwan Strait (WTPS), as shown in Figure 1. The surface sediment samples were collected using a Van Veen grab sampler (35 cm × 25 cm × 30 cm) at a depth of 0−5 cm. Samples from JRE (*n* = 16) were collected in January and February of 2019, and samples from the WTPS (*n* = 12) were collected in November and December of 2018. The sampling locations were identified accurately using a GPS locator. Furthermore, the collected sediment samples were stored in solvent-cleaned aluminum foil, sealed in polyethylene bags, placed on ice and transported to the laboratory, and frozen at −20 °C until further analysis. The physicochemical properties of the sediment overlying water were determined using YSI-EXO2 (Palo Alto, CA, USA), as shown in Table S2. The Jiulong River Basin is located in the southeast of Fujian Province, where the economy is relatively developed. The Jiulong River Basin has a total population of 5.43 million and a cultivated area of 1500 km^2^. The downstream Zhangzhou Plain covers an area of 567 km^2^. The Jiulong River flows through the dense industrial parks, agricultural areas, and aquaculture areas. Most of the production wastewater is discharged into the Jiulong River Basin, which causes great harm to the water quality of the Jiulong River, resulting in the increasingly serious pollution problem. As the receiving water body of the Jiulong River, the water quality of the Taiwan Strait is bound to be affected to some extent.

### 2.3. Sample Preparation and Instrumental Analysis

Samples were prepared following the method described by Li et al. [28]. Briefly, sediment samples were freeze-dried and sieved; approximately 0.5 g dry weight (dw) of each sample was spiked with an internal deuterated standard (d15-TPP). Then, an ultrasound-assisted extraction was performed thrice with 20 mL of an acetonitrile–water mixture (6:4). After centrifuging this mixture at 4000 rpm, ~40 mL of water was added to ~60 mL of the supernatant. Furthermore, the vial was filtered with a disposable 0.22 μm hydrophilic polytetrafluoroethylene (PTFE) syringe filter (Anpel Laboratory Technologies Inc. Shanghai, China). Solid-phase extraction (SPE) cartridges (Oasis HLB 200 mg, Waters, Milford, MA, USA) were conditioned with 4 mL of acetonitrile and 4 mL of Milli-Q water. After loading the sample at a rate of 0.5 mL/min, the cartridge was rinsed with 3 mL of Milli-Q water and vacuumed for 30 min until it was dry. Target compounds were eluted twice with 8 mL of acetonitrile (ACN). The combined eluate was passed under a gentle nitrogen flow to near dryness, reconstituted with 1 mL ACN, and filtered through a 0.22 μm PTFE syringe filter.

The analyses of target OFPRs were performed using an Agilent 1260 liquid chromatograph coupled to an Agilent 6460 triple quadrupole mass spectrometer (Palo Alto, CA, USA). The LC system was equipped with an Agilent EC-C18 (3.0 mm × 100 mm, 2.7 μm particle size) that was maintained at 35 °C. The mobile phases consisted of water (A) and ACN (B) at a flow rate of 0.5 mL/min. The following gradient was employed: 85% B ramped to 90% B in 2.50 min (linear), followed by a linear increase to 95% B in 1.75 min (held for 4.20 min), and then changed to 5% B for 4.08 min. Moreover, a 10 μL aliquot of the sample was injected into the LC system for analysis. The detailed LC-MS/MS analytical parameters for the determination of the target OPFRs are shown in Table S3.

### 2.4. Quality Assurance and Quality Control

This study applied a quality control procedure for data analysis [29]. D15-TPP was added to the samples as a surrogate to assess method recovery. Recovery of the surrogate was between 67% and 119%. The limit of detection (LOD) for each OPFR was defined as signal-to-noise (S/N) ratios of 3. Instrumental calibration standards were analyzed after every five samples to monitor instrument stability, and regression coefficients (R^2^) of the calibration curves were greater than 0.99.

### 2.5. Date Analysis

Using the sediment no-effect concentration (*PNEC*) as a water quality criterion to assess the ecological risk of OPFRs in the Jiulong River Estuary and Taiwan Strait, the predictions of five OPFRs were calculated using Equation (1) in combination with data from ECHA (European Chemicals Agency) [30].

(1) $$RQ=\frac{MEC}{PNEC}$$

where *RQ* is the ecological risk, and *MEC* is the actual concentration.

To assess the human exposure dose, OPFRs in sediments were converted to concentrations in the aqueous phase by multi-media imputation simulations, which were then converted to doses based on the USEPA exposure equation (USEPA, 2017). The equation is expressed as follows

(2) CW=CS×ρ 

(3) $$EXP_{D}=\frac{CW\times IR}{BW}$$

where *CS* is the concentration in sediment (ng/g), *ρ* is the conversion factor between the aqueous phase and sediment based on a provided, *CW* is the concentration in water (ng/L), *EXP_D_* is the exposure dose (ng/kg/bw/day), IR is the uptake rate (L/d), and *BW* is the body weight (kg). The *RQ* values for health risks were determined using the following equation.

(4) $$RQ=\frac{EXP_{D}}{RfD}$$

where *RfD* is the USEPA oral reference dose.

Spearman correlation analysis was performed using IBM SPSS statistical software (IBM, Armonk, NY, USA) for the pollutant-related parameters at both sites. Descriptive statistics (i.e., minimum, maximum, mean, and standard deviation) were used to analyze the relationship between residual concentrations.

## 3. Results and Discussions

### 3.1. Concentration of OPFRs in Sediments

As shown in Table 1 and Table 2, the concentration ranges of all OFPRs ranged between <LOD and 36.63 ng/g; furthermore, their concentration level was 10^2^ ng/g, which was high according to China’s standards [31]. Previous studies estimated that seven OPFRs in the sediments of Taihu Lake in China had concentration ranges of 3.4–14 ng/g, and five OPFRs in the southwest coast of Taiwan had concentration ranges of 1.0–13 ng/g; these values were lower than the values obtained in the present study [32,33]. Meanwhile, the concentration range in the Yangtze River was similar to the present study, ranging from 3.37 to 29.65 ng/g [34]. However, studies in the Pearl River Delta and in Spain found that OPFR concentrations could reach 10^3^ orders of magnitude, with maximum concentrations of 470 ng/g and 824 ng/g, respectively [17,33,35]. Of the five selected OPFRs, only TDCPP was not detected, probably due to local policies prohibiting the use of this compound.

Three of the four detected compounds, namely TCEP, TCPP, and EHDPP, had concentrations greater than 50%, indicating that they were more common in the sediments of the sampling sites. The detection rates of TCEP and TCPP in sediments obtained from the Yangtze River and Taihu Lake in China were close to 100% [7,34], and the detection rates of these two substances from the United States and Korea also reached 100%, 100% and 60%, 80% [36,37], respectively, indicating that their results were consistent with the present study. EHDPP in sediments is relatively less studied. Previous studies estimated that their detection rate in Korean sediments was 50%, while their detection rate in Guangxi, China, was only 16.7% [33,36]. Therefore, in this study, EHDPP may have a specific source of contamination. TDBPP studies tend to focus on water media because of the high detection rate of the substance in the water column, which was only 35.7% for sediments in this study [38].

The concentration distribution of the detected OFPRs was consistent with the detection rate. Detection rates of TCEP, TCPP, and EHDPP were one order of magnitude higher than those of TDBPP. The average concentration of EHDPP was 36.6 ng/g, which was significantly higher than its concentration in the sediment of Taihu Lake (ND−0.94 ng/g) [7]. EHDPP was reported to be more prominent in processed foods; thus, the surrounding population density and food industry could be important influencing factors [40]. Concentration ranges of TCEP and TCPP were similar and were consistent with previous reports on OPFRs [41,42].

### 3.2. Spatial Distribution of OPFRs in Sediments of the Jiulong Estuary and Western Taiwan Strait

**Figure 2:**
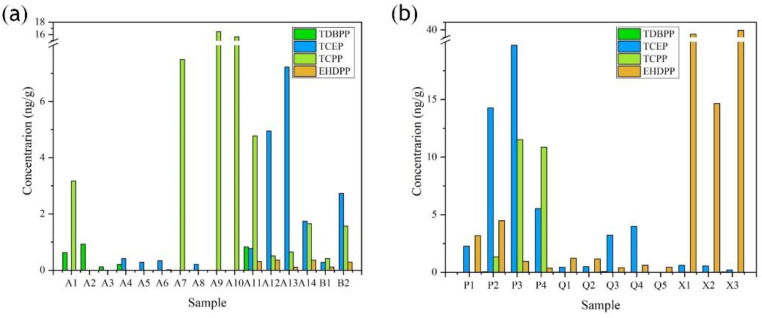
Distribution of OPFRs concentrations at sampling sites at (**a**) the Jiulong River estuary and (**b**) the western Taiwan Strait.

As seen in Figure 2, TDBPP, TCEP, and TCPP showed similar distribution characteristics in both regions, with their concentrations ranging from 0 to 20 ng/g.

The most obvious difference was observed in EHDPP, which were two orders of magnitude higher in the Taiwan Strait region than in the Jiulong region. Electronic waste is an important source of pollutants, and the Beijiang River basin upstream of the Jiulong River traverses the largest electronics factory waste recycling area in Guangzhou [33,43]. As a result, pollutant concentrations tend to be high in the Jiulong River basin. In addition, the neighboring city of Xiamen, which is highly developed, densely populated, and the industrial area, also plays a role. High concentrations of certain OPFRs were found in P_2_, P_3_, and P_4_. TCEP concentrations of 14.3 and 19.7 ng/g were found in P_2_ and P_3_, respectively, and TCPP exceeded 10^1^ orders of magnitude in P_3_ and P_4_. TCPP and TCEP are often used as flame retardants in industries that produce automotive, rubber, polyurethane foam (PUF), and textile coatings [17,44]. Pollutants from these industries may be discharged into water bodies and migrate into sediments [28].

TDBPP and TCPP were dominant in A_1_-A_3_, TCEP and TDBPP in A_4_-P_4_, and EHDPP and TCEP in Q_1_-X_3_. TCEP is being gradually replaced by TCIPP in many countries due to its carcinogenic and viscous nature [28]. The high level of TCEP in the sampling sites indicates its widespread use and low removal efficiency of the nearby wastewater treatment.

Several studies showed differences in OPFR concentrations in estuarine and coastal areas [17,28,45]. The concentrations generally show a decreasing trend from west to east owing to a strong dilution effect. When the pollutants reach the river–sea interface, they become disturbed by the mixing of freshwater and seawater and are deposited with the sediments at the boundary. The data for TOC, TN, and ammonia nitrogen in Table S2 also illustrate this phenomenon well. As the geographical location changes from west to east, the three data show a decreasing trend, especially for TOC, which decreases more significantly by one order of magnitude. The decrease in the concentrations of TDBPP, TCPP, and TCEP from the estuary to the coast was not obvious, indicating that river runoff was not their only source. However, the concentration of EHDPP showed a sharp increase in X_1_-X_3_ (marine area), which was much higher than the other points in the upper Taiwan Strait. This may be due to the input of coastal currents from winter to spring in Fujian and Zhejiang [28]. According to the relevant literature, industrial wastes discharged from coastal areas are a potentially important source of OPFRs in the ocean [46,47]. In addition, atmospheric deposition caused by the East Asian monsoon has an important influence on the spatial distribution of organic pollutants [28].

**Figure 3:**
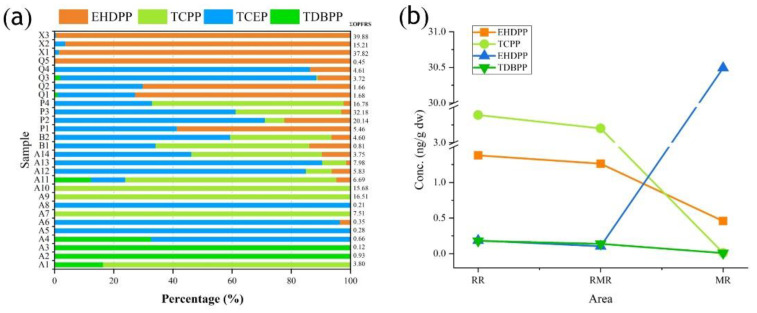
(**a**) Percentage distribution of OPFRs in the Jiulong River estuary and Taiwan Strait sampling sites; (**b**) Variation in OPFRs concentrations in different regions.

The spatial distribution of OPFRs in the Jiulong area may also be influenced by hydrological conditions. Watersheds can be roughly divided into three zones based on salinity gradients and local conditions [43]. The estuarine zone (RR, salinity < 5‰) is located at the end of the river and is mainly influenced by river runoff. The river–sea zone (RMR, 5‰ ≤ salinity ≤ 25‰) is influenced by both rivers and oceans according to the tidal cycle of the Taiwan Strait. The marine zone (MR, salinity ≥ 25‰), located at the end of the ocean, is heavily influenced by seawater. Based on geographical location and watershed division, sites A_1_-A_3_ are located in the RR, sites A_4_-A_14_ and B_1_-B_2_ in the MR, and sites X_1_-X_3_ are in the RMR. From Figure 3b, it can be seen that the distribution of the three OPFRs, except EHDPP, is consistent with salinity; that is, the distribution of OPFRs showed a decreasing trend with increasing salinity, which was similar to the result of previous studies [28]. It was reported that the large-sized particles in the RR zone were remobilized by deposition, while the small-sized particles were transported to the RMR and MR zones [28]. The small particles allowed OPFRs to occur mainly in water and suspensions, thus decreasing their concentrations in the sediments [7]. This indicated that the distribution of OPFRs in the sediments of the Jiulong River and Taiwan Strait was not only controlled by river runoff but also influenced by hydrodynamic, oceanic, and anthropogenic factors, which was consistent with the results of other studies [17,25,28].

### 3.3. Source Analysis of OPFRs

The correlations between the four OPFRs and total concentrations were analyzed using SPSS statistical software.

Table 3 show the correlation coefficients and significance levels. It was observed that the correlations among the OPFRs were weak. However, there was a good correlation between TCEP and TCPP, which indicated significant variability in their origin. This conclusion was consistent with the discussion in Section 3.2. Except for TDBPP, all three OPFRs were positively correlated with the total mean concentration. TCEP and TCPP were moderately correlated (0.3–0.5), whereas EHDPP had the most pronounced correlation of 0.769. Usually, a correlation coefficient between 0.5 and 0.8 is considered strong. Thus, EHDPP, TCEP, and TCPP were the highest contributors of OPFRs in the Jiulong River Estuary and Taiwan Strait.

**Figure 4:**
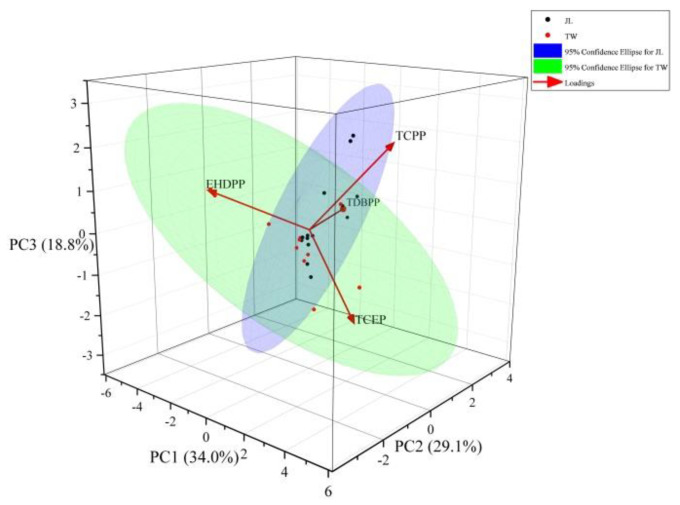
PCA master element analysis diagram. (JL: Jiulong River Estuary, TW: Taiwan Strait).

To further analyze the possible contaminant sources, principal component analysis (PCA) was performed on the parameters associated with the OPFRs. As seen in Figure 4, the three main factors, PC_1_, PC_2_, and PC_3_, had values of 34.0, 29.1, and 18.8%, respectively, accounting for 81.9% of the total variance. TCPP and TCEP had high loadings (>0.6) in PC_1_. Furthermore, they were reported to be highly enriched in the drinking water and surface waters of China, and their sources were closely related to emissions from vehicles and marine traffic [46,48,49]. Therefore, PC_1_ can be considered to be a combination of emissions from road runoff, vehicles, and marine traffic.

PC_2_ accounted for 29.1% of the total variance, with TDBPP (0.73907) having the highest value. TDBPP is often used as a flame retardant in cellulose, triacetate, and polyester fabrics [28]. It is commonly found in fabrics [17]. In addition, TDBPP solid waste from textile processing plants is an important source of pollution. Therefore, PC_2_ can be defined as a combination of emissions from industry and population.

The loads of EHDPP and TCPP in PC_3_ were more pronounced. Given that they are commonly present in the air, atmospheric particles, and dust [34], PC_3_ was a combination of the effect of atmospheric deposition and hydrology.

### 3.4. Risks of OPFRs

In this study, ecological risks were assessed based on risk quotients (RQs) calculated by the PNEC. The PNEC values provided by ECHA for TCPP, TCEP, TDBPP, and EHDPP are 292 ng/g, 200 ng/g, 92.9 ng/g, and 81.4 ng/g, respectively [50]. The risk assessment criteria were as follows: low risk (0.01 < RQ < 0.1), medium risk (0.1 ≤ RQ < 1), and high risk (RQ ≥ 1).

**Figure 5:**
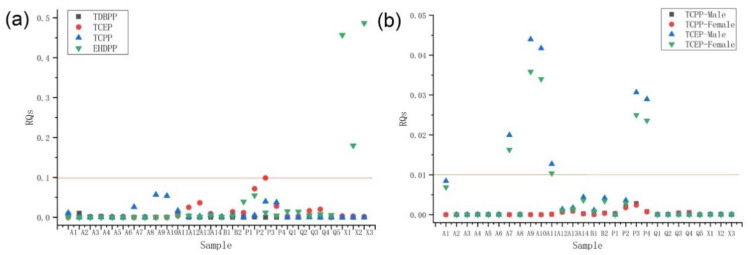
(**a**) Ecological risk assessment of all sampling sites; (**b**) Health risk assessment of all sampling sites.

Figure 5a show that all detected OPFRs have low or no risk at the sampling points. TCEP is almost risk-free at most sampling sites and is a low risk only in zone Q_4_, with a value of 0.0199. TDBPP is similar, reaching 0.01 only in zone A_2_. TCPP has a more balanced risk distribution, ranging from 0 to 0.0565, and generally has a low risk. In contrast, the ecological risk of EHDPP was more severe, with three areas falling into the medium risk zone. The highest risk values for EHDPP were found in the X zone, with 0.457, 0.180, and 0.487, respectively, which were far from the risk values of the other three substances. This was consistent with the results of the study on Taihu Lake [7]. Moreover, the risk of pollutant coexistence in the basin should not be underestimated. The high risk of certain substances increases the risk value of the total concentration. Thus, there is a need for long-term detection and control when the overall risk level in the two zones is high.

As shown in Table S4, the high water solubility of OPFRs in this study suggests that most of these compounds tend to be distributed in the aqueous phase. Therefore, the higher ecological risk of TCEP, TCPP, and EHDPP in sediments suggests that these compounds are also at high risk in water. In addition, the studied watersheds are also used for drinking water and fisheries, and OPFRs in sediments can enter humans indirectly through drinking water and organisms, which has important implications for human exposure and human health and therefore requires further assessment of human health risks [30]. At the same time, the sensitivity analysis of the organism is an important parameter for subsequent studies when conducting risk assessments.

The theoretical health risks of OPFR in adult males and females in this study were analyzed using the relevant formulae (Figure 5b). Since there were no other RfD reference values for OPFR, only the health risks for TCPP and TCEP were calculated. The RfD values for TCPP and TCEP were 10ng/g/day and 7ng/g/day, respectively, with conversion factors ρ of 0.0412 and 0.544 [51,52]. The remaining parameters are shown in Table S5. As can be seen in Figure 5b, most of the sampling sites had almost no risk. Only the TCPP located in parts A_7_-P_4_ had low risk with the highest value of 0.0439. In addition, the risk values were generally higher for males relative to females. In conclusion, the OPFR in both the Jiulong River Basin and the Taiwan Strait have low health risks but are still not negligible.

## 4. Conclusions

In this study, OPFRs were detected and analyzed in the sediments obtained from the Jiulong River Basin and the Taiwan Strait; their concentrations, frequencies, spatial distributions, and ecological risk profiles were systematically described. Four of the five OPFRs were detected in the samples. Furthermore, TCPP, TCEP, and EHDPP were the major contaminants, given their high frequency of use. The spatial distribution of OPFRs was mainly influenced by river runoff, monsoon, and hydrology. Correlation and PCA showed that the sources of OPFRs were multifaceted, as follows: (1) emissions from road runoff and surface traffic, (2) influence of atmospheric deposition and hydrological conditions, and (3) a combined effect of industry and population. The ecological and health risks indicate that the overall risk values of the OPFR are not negligible, despite the low individual risk values. This study can facilitate the development and implementation of future pollution control strategies for the relevant sectors in two selected regions and provide data support for global pollutant studies.

## Figures and Tables

**Table 1:** Concentrations and frequencies of the five OPFRs in the sediments of Jiulong River Estuary and adjacent Western Taiwan Strait.

OPFRs	Min	Max	Range	Med	Average	SD	DF%
TDBPP	0.00	0.926	<LOD-0.926	0.0971	0.289	0.343	35.7
TCEP	0.00	19.7	<LOD-19.7	0.771	3.34	4.92	75.0
TCPP	0.00	16.5	<LOD-16.5	1.61	4.48	5.55	60.7
EHDPP	0.00	36.6	<LOD-36.6	0.448	5.57	11.7	67.9
TDCPP	NA	NA	<LOD	NA	NA	NA	0.00
∑OPFRs	0.118	39.9	-	4.61	9.12	11.2	-

**Table 2:** Global concentrations of OPFRs (ng/g).

Region	TDBPP	TCEP	TCPP	EHDPP	Year	Reference
Yangtze River	-	3.13–4.08	3.37–29.7	-	2018	[34]
Taihu Lake	ND	1–3.17	0–2.19	ND-0.94	2018	[7]
Qinzhou Bay	-	0–3.076	-	0–94.4	2021	[39]
Pearl River Delta	-	ND-58	0.91–185	-	2016	[35]
United States	-	0.168–5.6	0.146–36.8	-	2016	[37]
Australia	-	0–160	33–170	-	2021	[17]
Korea	-	0–60	0–216	0–50	2018	[36]

**Table 3:** Correlation analysis between the components as well as total concentration.

	TDBPP	TCEP	TCPP	EHDPP	∑OPFRs
TDBPP	1.000	−0.177	−0.054	−0.134	−0.193
TCEP	−0.177	1.000	0.218	−0.100	0.405 *
TCPP	−0.054	0.218	1.000	−0.194	0.348
EHDPP	−0.134	−0.100	−0.194	1.000	0.769 **
∑OPFRs	−0.193	0.405 *	0.348	0.769 **	1.000

Note. “**”, Significant correlation at *p*  <  0.01; “*”, significant correlation at *p*  <  0.05.

## Data Availability

All data generated and analyzed during this study are included in this published article and its supplementary information files.

## Supplementary Materials

The following supporting information can be downloaded at: https://www.mdpi.com/article/10.3390/ijerph19042449/s1, Table S1: Structures and physicochemical properties of the target organophosphate flame retardants, Table S2: Sediment sampling information, Table S3: LC-MS/MS analytical parameters for determination of the target organophosphate flame retardants, Table S4: Acute and chronic HC5 of five OPFRs, Table S5: The parameters for health risk assessment.
