# Supplementary material for: Occurrence, Distribution, and Risk of Organophosphate Flame Retardants in Sediments from Jiulong River Estuary and Adjacent Western Taiwan Strait, China
Source: Int J Environ Res Public Health. 2022 Feb 20;19(4):2449. doi: 10.3390/ijerph19042449 (PMC8872513; doi:10.3390/ijerph19042449)
Supplement: Supplementary file 1 [file ijerph-19-02449-s001.zip › ijerph-1556286-supplementary.pdf]

Supplementary Materials

Table S1. Structures and physicochemical properties of the target organophosphate flame retardants

Compound Name	Abbreviation	CAS NO.	Structure	Molecular Formula	Molecular Weight	Log K_{ow}[a]	BCF[a]
2-Ethylhexyl diphenyl phosphate	EHDPP	1241-94-7	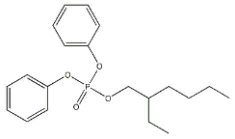	$C_{20}H_{27}O_4P$	362.4	5.73	309.03
Tris(2,3-dibromopropyl) phosphate	TDBPP	126-72-7	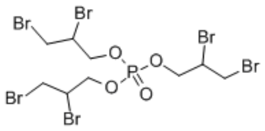	$C_9H_{15}Br_6O_4P$	697.6	4.29	3.24
Tris(1,3-dichloroisopropyl) phosphate	TDCPP	13674-87-8	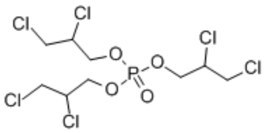	$C_9H_{15}Cl_6O_4P$	430.9	3.65	12.02
Tris (2-chloroisopropyl) phosphate	TCPP	13674-84-5	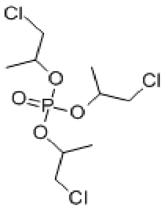	$C_9H_{18}Cl_3O_4P$	327.6	2.59	7.94
Tris(2-chloroethyl) phosphate	TCEP	115-96-8	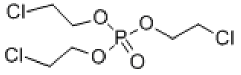	$C_6H_{12}Cl_3O_4P$	285.5	1.44	1.29

Log K_{ow}: octanol-water partition coefficient; BCF: bioconcentration factor. a. The data are from EPA DSSTox (https://comptox.epa.gov/)

Table S2. Sediment sampling information

Regions	Station Name	Latitude	Longitude	Depth (m)	Temperature (℃)	Salinity (ppt)	pH	Chla (ug/L)	ODO (mg/L)	BGA-PE (μg/L)	TDS (mg/L)	NH^{3+}-N (mg/kg)	TOC (mg/kg)	TN (mg/kg)
Jiulong	A1	24.48	117.78	7.8	18.10	2.36	7.77	3.45	6.07	8.07	2866	120.51	1279.86	154.90
River	A2	24.47	117.79	7.6	17.83	4.23	7.75	4.18	6.24	9.94	4954	108.61	1158.20	129.28
Estuar	A3	24.46	117.80	11	17.58	6.57	7.64	3.97	6.56	9.60	7460	105.10	1101.47	116.53
y	A4	24.49	117.82	9.3	17.36	9.34	7.72	3.41	6.93	9.53	10337	97.45	1195.43	130.39
(JRE,	A5	24.43	117.84	9.8	17.04	13.35	7.75	2.67	7.37	7.67	14366	57.51	1166.27	109.17
n=16)	A6	24.43	117.85	6.9	17.06	13.20	7.73	2.33	7.21	7.00	14215	22.93	241.16	3.97
	A7	24.41	117.88	6.9	17.18	13.88	7.79	2.77	7.32	8.30	14891	62.07	1133.88	102.68
	A8	24.41	117.89	5.8	17.31	14.06	7.75	2.65	7.35	7.86	15067	18.54	670.16	49.33
	A9	24.40	117.91	5.7	17.56	15.13	7.80	2.84	7.73	7.58	16113	34.17	711.70	52.54
	A10	24.40	117.93	6.6	17.17	17.91	7.98	2.41	7.83	6.41	18797	119.54	1208.42	127.23
	A11	24.40	117.97	7.2	17.04	21.05	7.99	1.86	7.99	5.78	21777	10.03	842.85	62.26
	A12	24.41	118.00	9.5	16.95	23.17	8.02	2.04	8.11	5.59	23766	18.03	903.66	101.76
	A13	24.42	118.03	10.0	16.84	25.66	8.04	1.82	8.1	5.50	26067	52.02	890.22	91.74
	A14	24.42	118.06	10.5	16.66	26.62	8.05	1.90	8.12	6.08	26947	48.19	1327.65	95.20
	B1	24.43	117.91	11.0	17.13	26.28	8.11	2.28	8.07	6.60	26632	55.35	914.52	79.06
	B2	24.45	117.93	4.5	17.24	26.61	8.11	2.13	8.16	5.84	26928	24.07	941.82	118.74
Western	Q1	25.01	119.05	31.8	21.17	30.82	8.07	1.00	7.41	3.45	30747	57.37	517.57	54.87
Taiwan	Q2	24.78	118.81	19	21.22	30.65	8.12	1.11	7.41	3.40	30594	24.68	512.55	63.37
Strait	Q3	24.71	118.92	31.7	21.72	31.01	8.14	1.01	7.42	3.46	30919	32.91	174.52	16.80
(WTS,	Q4	24.61	119.12	57.6	23.18	33.14	8.18	1.15	7.20	4.10	32838	25.88	224.78	36.10
n=12)	Q5	24.50	119.64	47	24.74	34.42	NA	NA	NA	NA	NA	24.27	732.88	91.56

P1	25.51	120.12	51	23.17	33.95	NA	NA	NA	NA	NA	23.20	561.17	74.71
P2	25.18	119.40	24	19.85	30.17	NA	NA	NA	NA	NA	16.27	843.42	117.09
P3	25.09	119.57	34	20.38	30.68	NA	NA	NA	NA	NA	15.54	684.25	88.15
P4	25.02	119.78	76	23.31	34.08	NA	NA	NA	NA	NA	16.89	192.21	28.09
X1	24.41	118.10	16.5	22.16	30.04	7.97	1.39	6.92	5.20	30054	30.81	862.88	148.41
X2	24.29	118.29	19	20.84	31.04	NA	NA	NA	NA	NA	17.71	614.53	87.14
X3	24.21	118.48	44	21.25	32.24	NA	NA	NA	NA	NA	16.34	853.04	84.37

Cond, TDS, ORP, pH water, DO were measured in the bottom water; Chla was measured in the surface water; pH sediment, TOC, TN, TP were measured in the surface sediment.

Table S3. LC-MS/MS analytical parameters for determination of the target organophosphate flame retardants

Compound	Molar mass (g/mol)	ESI	Precursor (m/z)	Produc t (m/z)	Retention time (min）	Cone voltage (V)	Collision voltage (V)	R^2	S/N	LOQ
EHDPP	362.4	Negtive	363.1	250.9	6.52	110	6	0.9995	1371.7	0.106
TDBPP	697.6	Negtive	698.6	99	5.56	150	26	0.9999	282.9	0.160
TDCPP	430.9	Negtive	430.9	99	5.37	145	24	0.9995	187.1	0.021
TCPP	327.6	Negtive	327.1	99	4.98	110	18	0.9957	694.9	0.043
TCEP	285.5	Negtive	284.9	63.1	4.52	130	25	0.9974	291.1	0.103
d15-TPP	342	Negtive	342	162	4.45	155	28	0.9965	352.7	0.059

Table S4. Acute and chronic HC5 of five OPFRs

		TCEP (mg/L)	TDBPP (mg/L)	EHDPP (mg/L)	TCPP (mg/L)	TDCPP (mg/L)
Acute	EXT2.0	2.96	0.21	0.02	-	0.52
	Sigmaplot3.0	3.74	0.18	0.05	-	0.58
Chronic	EXT2.0	0.03	0.16	0.01	0.05	-
	Sigmaplot3.0	0.04	0.22	0.02	0.09	-

('-' means lack of data)

Table S5. The parameters for health risk assessment.

	Male	Female
IR (L/day)	2.23	1.65
BW (kg)	65	56
EF (day year-1)	365	365
